# First Case of H Syndrome in Northern Morocco: Clinical Features and Literature Overview

**DOI:** 10.2478/bjmg-2026-0004

**Published:** 2026-10-14

**Authors:** H Jelti, A Lamaibdel, S Bouressas, M Benkacem, S Regragui, A Lamzouri

**Affiliations:** Department of Medical Genetics and Oncogenetics, Mohammed VI University Hospital, Tangier, Morocco; Life and Health Sciences Laboratory, Faculty of Medicine and Pharmacy of Tangier, Abdelmalek Essaâdi University,Morocco; Department of endocrinology, Mohammed VI University Hospital, Tangier, Morocco; Department of Hematology, Mohammed VI University Hospital, Tangier, Morocco

**Keywords:** H syndrome, hyperpigmentation, hypertrichosis, insulin-dependent diabetes mellitus, *SLC29A3*

## Abstract

H syndrome is a rare multisystem genodermatosis with an autosomal recessive inheritance, characterized by cutaneous hyperpigmentation, hypertrichosis, hepatosplenomegaly, hearing loss, heart anomalies, hypogonadism, short stature, hyperglycemia, hallux valgus and flexion contractures of fingers and toes. To date, 161 cases have been reported worldwide with limited data from North Africa. Herein, we report the case of a 17-year-old Moroccan girl diagnosed with H syndrome on the basis of clinical features and molecular testing. Clinical examination revealed dysmorphic features with insulin-dependent diabetes, sensorineural hearing loss, short stature, and progressive hyperpigmented indurated skin lesions with hypertrichosis predominantly affecting the thighs and genital region, associated with flexion contractures of the fingers and toes. Genetic testing identified a homozygous pathogenic splice-site variant c.300+1G>C in the *SLC29A3* gene, previously reported to be associated with H syndrome. This is the first report of this mutation from northern Morocco, highlighting regional prevalence, phenotypic variability and the challenges faced in diagnosis due to high cost and limited access to molecular testing.

H Syndrome (OMIM#602782) is a rare multisystem genodermatosis with autosomal recessive inheritance. Since its first description in 2008 by Molho-Pessach et al[1], more than 160 cases have been reported worldwide. The manifestations of H syndrome include characteristic cutaneous findings, notably hyperpigmentation associated with hypertrichosis and progressive sclerodermatous induration, typically involving the medial thighs while sparing the knees. Additional systemic features include hepatosplenomegaly, hearing loss, heart anomalies, hypogonadism, low height (short stature), hyperglycemia (insulin-dependent diabetes mellitus) and skeletal abnormalities such as hallux valgus/flexion contractures of fingers and toes[2].

H syndrome results from mutations in the *SLC29A3* gene, which is located on chromosome 10q22 and encodes the human equilibrative nucleoside transporter 3 (hENT3)[3].

Although many mutations have been reported since the initial description, a solid genotype–phenotype correlation is lacking. In this report, we present the first case in northern Morocco.

## MATERIALS AND METHODS

### Clinical Report

A 17-year-old Moroccan female, the older of two siblings, was referred to genetics clinical counseling for evaluation of short stature. She was born to related parents, with a birth weight of 3300 g and normal psychomotor development.

She has been followed since the age of 5 for insulin-dependent diabetes, managed with a basal bolus regimen, and for bilateral progressive sensorineural hearing loss for which she has been prescribed hearing aids.

Furthermore, the patient developed progressive flexion contractures of the fingers and toes. She also presented with symmetrical and bilateral indurated hyperpigmented skin lesions associated with hypertrichosis, initially involving the thighs. These lesions progressively extended to the genital, inguinal, abdominal, and lumbar regions, while sparing the knees, buttocks, upper back, and chest (Figure 1).

**Figure 1.**
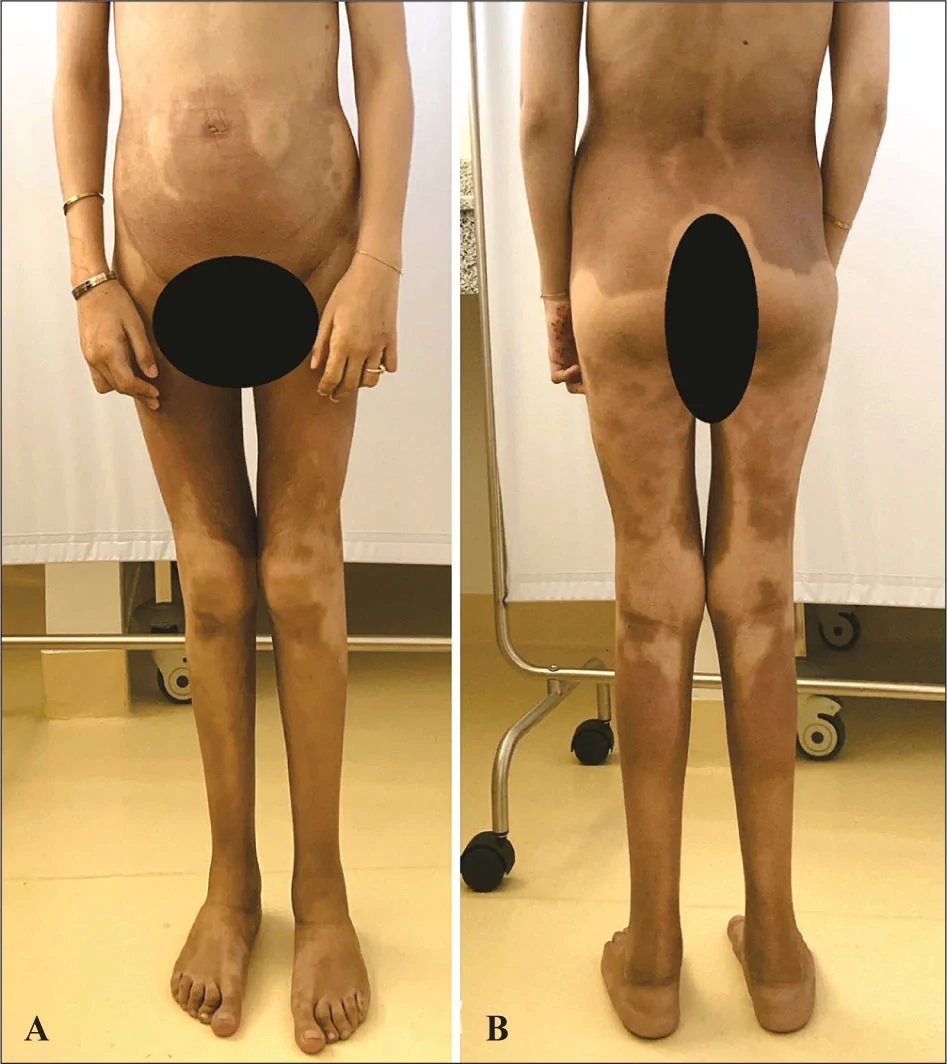
Hyperpigmentation and hypertrichosis patches over the inner thighs, the genitalia, the inguinal, the abdominal and the lumbar areas sparing the knees, buttocks, upper back and the chest.

At the age of 8, the proband presented abdominal, mediastinal, and inguinal lymphadenopathies. A biopsy was performed, revealing a histological aspect suggestive of nonspecific hyperplastic lymphadenopathy. Subsequently, the patient developed a hypogonadism with undeveloped pubertal signs.

The clinical examination revealed a stature significantly below average, measuring 140 cm (−4 SD), a weight of 32 kg (−3 SD) (body mass index 16.32 kg/m^2^) and a head circumference of 55 cm (within the normal range). Additionally, the patient had dysmorphic features including an oval face, wide forehead, thick eyebrows with synophrys, bilateral epicanthus, short palpebral fissures, bilateral corneal arcus, depressed nasal bridge, infiltrated cheeks and a deep philtrum.

Examination of extremities revealed camptodactyly of the 4th and 5th fingers bilaterally, involving the proximal interphalangeal joints (Figure 2), as well as hallux valgus and bilateral flexion contracture of the 2nd toes. No hepatosplenomegaly or cardiac abnormalities were noted.

**Figure 2.**
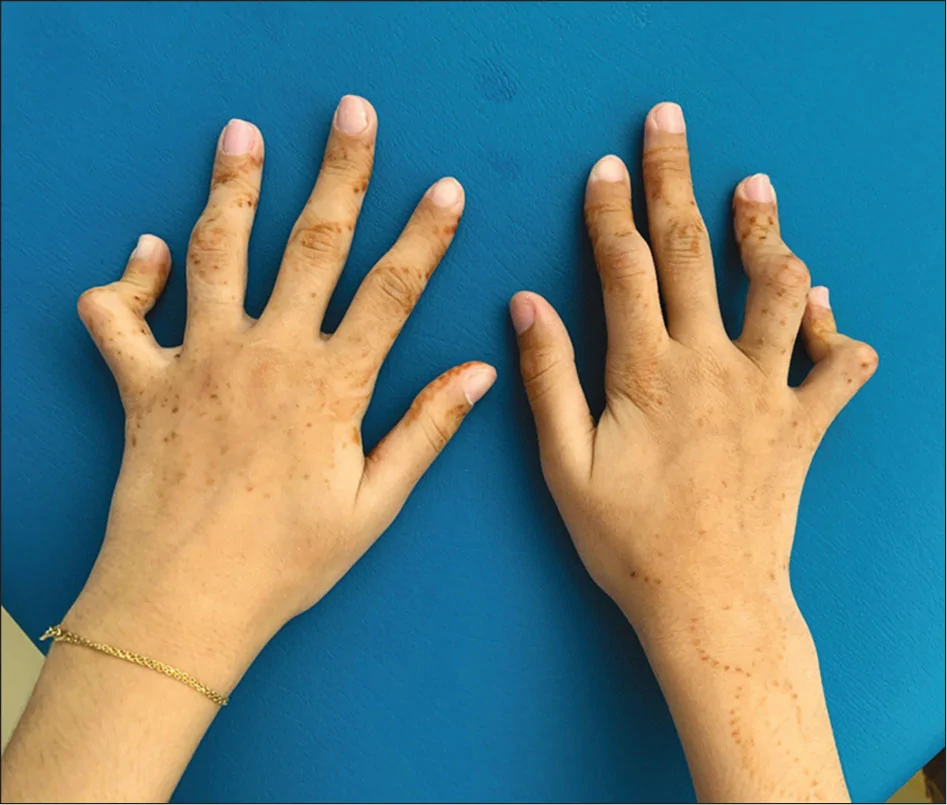
Fixed flexion contractures of the proximal interphalangeal joints of the 4th and 5th fingers bilaterally.

Laboratory findings included mild microcytic hypochromic anemia (Hemoglobin 8.7g/dl) with normal ferritin level, intermittent elevation of C-reactive protein (CRP) with an elevated glycated hemoglobin at 7.6%. Insulin-like growth factor 1 (IGF-1) level was also found to be low at 43.1 ng/ml with the presence of Anti-glutamic acid decarboxylase (anti-GAD) antibodies.

No other cases have been reported in the family.

Based on these clinical and paraclinical observations, H syndrome was suspected.

### Genetic testing

DNA was extracted from peripheral blood collected from the proband. A panel of 110 genes implicated in hearing loss was performed on the NextSeq500 Illumina platform. PolyWeb interface with Polyphen-2 HumanVar, SIFT, CADD, MutationTaster, MaxEntScan, SSF-like, NNSplice and GeneSplicer were used to perform bioinformatic predictions of mutation effects.

Written informed consent was obtained from the patient’s parents for genetic testing and publication.

## RESULTS

A pathogenic variant in the *SLC29A3* gene was identified in the homozygous state: (NM_018344.4: c.300+1G>C) (p.?). This variant affects the canonical splice site of intron 2 and is predicted to result in aberrant splicing. According to the American College of Medical Genetics and Genomics (ACMG) guidelines, it was classified as pathogenic, based on the following criteria: PVS1, PM2, PP3. This variant has been previously reported in patients with H syndrome.

Parental segregation analysis was not performed due to limited availability of testing.

## DISCUSSION

Histiocytosis represent a heterogeneous group of rare disorders characterized by an abnormal accumulation of histiocytes with enhanced phagocytic activity. Despite increasing recognition, their underlying pathophysiology remains incompletely understood, and therapeutic approaches are largely non-specific.

Within this group, the histiocytosis–lymphadenopathy plus syndrome encompasses a spectrum of clinically overlapping conditions that were previously considered distinct entities. These include Faisalabad histiocytosis (FHC), sinus histiocytosis with massive lymphadenopathy (SHML), also known as familial Rosai–Dorfman disease, H syndrome, and pigmented hypertrichosis with insulin-dependent diabetes mellitus (PHID). Accumulating evidence has demonstrated that these disorders share common phenotypic features and arise from mutations in the same gene, leading to their reclassification as a single disease spectrum.

All forms within this spectrum are caused by homozygous or compound heterozygous mutations in *SLC29A3*. Consequently, they are collectively referred to as *SLC29A3* spectrum disorders. This genetic unification explains the considerable clinical overlap observed among patients, including variable combinations of systemic and cutaneous manifestations.

The term “H syndrome” was first introduced by Pessach et al. following the description of ten patients from consanguineous Arab families. The designation reflects the constellation of characteristic features, many beginning with the letter “H,” including hyperpigmentation, hypertrichosis, hepatosplenomegaly, heart anomalies, hearing loss, hypogonadism, short stature (low height), and, in some cases, hyperglycemia. Together, these findings illustrate the broad and multisystemic nature of *SLC29A3*-related disease, forming the basis for current understanding of this unified histiocytic disorder spectrum[2]. The most frequently reported signs comprise cutaneous hyperpigmentation, hypertrichosis, and induration, predominantly affecting the thighs, genitalia, inguinal region, back, and abdomen. Subsequently, flexion contracture of the fingers and toes emerges as a common manifestation, followed by sensorineural hearing loss. In our case, we observed these signs as consistent manifestations, while other symptoms, including short stature were less frequent. Additionally, we noted swollen cheeks, a rarely reported manifestation resulting from histiocytic infiltration of subcutaneous tissue[4], evident from comparisons with old photographs of our patient.

Hematological investigations to evaluate the anemia are ongoing. So far, tests show elevated inflammatory markers, suggesting an underlying inflammatory or immune-related process. Reported abnormalities include reduced red blood cell production (red cell aplasia and dyserythropoiesis) and bone marrow changes such as myelofibrosis. There is also evidence of increased red blood cell destruction, with hemolytic anemia and a positive direct Coombs test. Additionally, pancytopenia and severe anemia with low reticulocyte counts have been noted, along with a nonclonal myeloproliferative pattern marked by increased monocytes and histiocytes in the bone marrow [1].

Although skin pathology report of our patient was unavailable, previous histopathological analysis in H syndrome revealed epidermal thickening, increased melanin deposition, dermal fibrosis, and CD68^+^ histiocytic infiltrates resembling the immunophenotype Rosai–Dorfman disease (RDD)[5]. A Recent series revealed diverse findings. Out of the 10 samples examined, three exhibited characteristic Rosai-Dorfman disease (RDD) histology, six displayed unclassified histiocytosis, and one showed a mixed pattern of RDD and Langerhans cell histiocytosis (RDD-LCH)[6].

The *SLC29A3* gene, located on chromosome 10q22, encodes the human *Equilibrative Nucleoside Transporter 3* (hENT3), this protein consists of 475 amino acids, involved in the transport of nucleosides and deoxynucleosides across lysosomal and mitochondrial membranes[3,7, 8, 9]. This sustains an intracellular reservoir of nucleosides necessary for various cellular pathways[8]. At the organ level, the gene is highly expressed in placenta, uterus, ovary, spleen, lymph nodes and bone marrow[3].

H syndrome can be attributed to various genetic mutations in the *SLC29A3* gene, encompassing missense, nonsense, compound, or deletion mutations. These diverse genetic alterations likely underlie the significant variability observed between different affected families. As the time of writing this manuscript a total of thirty-two pathogenic or likely pathogenic variants have been reported in the public database ClinVar[10], these mutations are thought to hinder nucleosides transport, leading to its accumulation inside lysosomes[11].

The c.300+1G>C mutation found in our patient is one of this *SLC29A3* pathogenic variants linked to H syndrome. It is a splice-site mutation known to disrupt the canonical donor site in intron 2, leading to aberrant splicing and the production of a truncated protein[12]. This mutation has been previously reported in two Egyptian and one Moroccan patients in a homozygous state, and in another compound heterozygous Moroccan case with H syndrome[ 12, 13, 14]. These previously reported cases had severe clinical features, such as insulin-dependent diabetes, hyperpigmentation, and multiple organ involvement, closely aligning with the symptoms observed in our patient and may suggest a potential genotype-phenotype correlation (Table 1), where mutations at this critical splice site may affect the severity and expression of the disease[14].

**Table 1:** Comparison of clinical, biological, and molecular features of reported cases of *SLC29A3*-related disorder (H syndrome) harboring the splice-site variant c.300+1G>C, including the present case.

**Clinical Feature**	**Present case**	**Bakhchane et al. (2016)**	**Elbarbary et al. (2013)**	**Elbarbary et al. (2013)**	**de Jesus et al. (2013)**
**DEMOGRAPHICS**					
Geographical origin	Morocco	Morocco	Egypt	Egypt	Morocco
Gender	Female	Female	Female	Male	Female
Age at diagnosis	17 years	13 years	19 years	15 years	12 years
**CUTANEOUS FEATURES**					
Hyperpigmentation	+	+	+	+	+
Hypertrichosis / sclerosis	+	+	−	NR	NR
Distribution of skin lesions	Genital areas, thighs, abdomen, lumbar region	Thighs, genital areas	Lower half of body	Cheeks and nose	Thighs
**MUSCULOSKELETAL**					
Flexion contractures / hallux valgus	+	NR	+	+	+
Short stature	+ (−4 SD)	+ (NR)	+ (−4 SD)	+ (−3 SD)	+ (−3 SD)
**ENDOCRINE MANIFESTATIONS**					
Insulin-dependent diabetes mellitus	+	−	+	NR	+
Hypogonadism	+	−	+	−	−
**NEUROLOGICAL & SENSORY**					
Sensorineural hearing loss	+	+	+	+	NR
Cognitive development	Normal	NR	Mild intellectual disability (IQ 65)	Low-normal range	NR
**SYSTEMIC INVOLVEMENT**					
Hepatosplenomegaly	−	−	+	−	+
Lymphadenopathy	+	NR	+	−	−
Anaemia	+	NR	NR	NR	+
Elevated inflammatory markers	+	+	+	+	−
Echocardiogram findings	NA	Normal	Pericardial effusion; concentric LV hypertrophy	Normal	NA (cardiomegaly on chest X-ray)
**HISTOPATHOLOGICAL FINDINGS**					
Skin/ lymph node biopsy	Hyperplastic lymphadenopathy	Perivascular Inflammatory infiltrate (histiocytes, lymphocytes); thickened collagen bundles in hypodermis	Mild perivascular lymphocytic infiltrate	NA	Hyperpigmentation of basal cell layer
**MOLECULAR GENETICS**					
*SLC29A3* variant (NM_018344)	c.300+lG>C (hom.)	c.300+1G>C and c.243delA (compound het.)	c.300+1G>C (hom.)	c.300+1G>C (hom.)	c.300+1G>C (hom.)

**Abbreviations:** +: present; –: absent; NR: not reported; NA: not available; F: female; M: male; SD: standard deviation; LV: left ventricular; IQ: intelligence quotient; hom.: homozygous; het.; heterozygous.

Functional studies have demonstrated disruptions in the lysosomal nucleoside trafficking system in ENT3 knockout mice, highlighting the crucial role of ENT3 as a transmembrane transporter essential for maintaining lysosomal integrity, causing inefficient clearance of debris of apoptotic cells in tissues and increased hematopoietic growth factors signaling, which leads to elevated number of macrophages and histiocytes [11,15].

The timely implementation of molecular testing was significantly hindered by its unavailability and prohibitive costs within our current setting, a limitation that proved particularly consequential in the context of autosomal recessive disorders. This challenge cannot, however, be viewed in isolation from the broader epidemiological landscape of our region. Consanguinity represents a major and historically entrenched predisposing factor to autosomal recessive disease in North Africa, and Morocco is no exception. National estimates place the prevalence of consanguineous unions at approximately 20–25%, with substantial regional variation [16]. From a public health perspective, this confluence of diagnostic inaccessibility and elevated genetic risk imposes a considerable and largely underestimated burden on an already under-resourced healthcare infrastructure, encompassing repeated hospitalizations, multidisciplinary care, long-term disability support, and the cumulative socioeconomic cost borne by affected families. Addressing this gap requires coordinated efforts at multiple levels: reinforcing molecular diagnostic capacity through subsidized national rare diseases programs, raising public awareness about the risks of consanguineous unions, and actively involving civil society organizations and patient associations in community outreach. Further research is also needed to better understand the social, economic, and cultural drivers that sustain consanguinity in the Moroccan context, as evidence-based insight is a prerequisite for any effective and culturally informed public health response.
